# Assessment of Probiotic Viability during Cheddar Cheese Manufacture and Ripening Using Propidium Monoazide-PCR Quantification

**DOI:** 10.3389/fmicb.2012.00350

**Published:** 2012-10-04

**Authors:** Émilie Desfossés-Foucault, Véronique Dussault-Lepage, Clémentine Le Boucher, Patricia Savard, Gisèle LaPointe, Denis Roy

**Affiliations:** ^1^Département des Sciences des aliments et de nutrition, Institut des nutraceutiques et des aliments fonctionnels, Université LavalQuebec, QC, Canada; ^2^École Supérieure d’Agriculture (Groupe ESA)Angers, France

**Keywords:** probiotic viability, Cheddar cheese, propidium monoazide, quantitative PCR, lactococci

## Abstract

The use of a suitable food carrier such as cheese could significantly enhance probiotic viability during storage. The main goal of this study was to assess viability of commercial probiotic strains during Cheddar cheesemaking and ripening (4–6 months) by comparing the efficiency of microbiological and molecular approaches. Molecular methods such as quantitative PCR (qPCR) allow bacterial quantification, and DNA-blocking molecules such as propidium monoazide (PMA) select only the living cells’ DNA. Cheese samples were manufactured with a lactococci starter and with one of three probiotic strains (*Bifidobacterium animalis* subsp. *lactis* BB-12, *Lactobacillus rhamnosus* RO011, or *Lactobacillus helveticus* RO052) or a mixed culture containing *B. animalis* subsp. *lactis* BB-12 and *L. helveticus* RO052 (MC1), both lactobacilli strains (MC2), or all three strains (MC3). DNA extractions were then carried out on PMA-treated and non-treated cell pellets in order to assess PMA treatment efficiency, followed by quantification using the 16S rRNA gene, the elongation factor Tu gene (*tuf*) or the transaldolase gene (*tal*). Results with intact/dead ratios of bacteria showed that PMA-treated cheese samples had a significantly lower bacterial count than non-treated DNA samples (*P* < 0.005), confirming that PMA did eliminate dead bacteria from PCR quantification. For both quantification methods, the addition of probiotic strains seemed to accelerate the loss of lactococci viability in comparison to control cheese samples, especially when *L. helveticus* RO052 was added. Viability of all three probiotic strains was also significantly reduced in mixed culture cheese samples (*P* < 0.0001), *B. animalis subsp. lactis* BB-12 being the most sensitive to the presence of other strains. However, all probiotic strains did retain their viability (log 9 cfu/g of cheese) throughout ripening. This study was successful in monitoring living probiotic species in Cheddar cheese samples through PMA-qPCR.

## Introduction

A minimum of 10^7^ cfu/mL or g and even higher numbers such as 10^8^ and 10^9^ cfu per daily portion of probiotic bacteria must be present in a product to retain their health benefits (Gomes da Cruz et al., 2009; Bhadoria and Mahapatra, 2011; Karimi et al., 2011). The use of an appropriate food carrier is thought to help promote bacterial viability and stability during storage (Roy and Delcenserie, 2011). Probiotic Cheddar cheese has already been successfully manufactured for this purpose (Daigle et al., 1999; Phillips et al., 2006; Ong et al., 2007) and suitable probiotic bacteria should be carefully chosen for this type of delivery vehicle.

As lactobacilli species are naturally found in this type of cheese and are resistant to acid production during cheese manufacture, some strains of *Lactobacillus acidophilus*, *Lactobacillus casei*, *Lactobacillus rhamnosus*, and *Lactobacillus plantarum* have a technological advantage for use as probiotics. Among the bifidobacteria in use, *Bifidobacterium animalis* subsp. *lactis* BB-12 is an especially acid resistant and aerotolerant strain, making it suitable for applications in such a food matrix (Roy et al., 2011). As each strain has their own technological properties and possesses specific health benefits (Roy, 2011), a combination of strains could also enhance the health value of the product (Temmerman et al., 2004). Furthermore, interactions between probiotic strains, but also with the lactococci Cheddar cheese starter, could affect bacterial viability in cheese samples (Gomes and Malcata, 1999; Ong et al., 2007).

Probiotic viability is usually monitored with traditional culture-dependent methods, which are both time consuming and imprecise. They can underestimate microbial counts and not detect viable but non-cultivable (VBNC) bacteria or lack specificity, especially for the closely related lactic acid bacteria found in cheese (García-Cayuela et al., 2009; Ndoye et al., 2011). Bacterial quantification is now possible through quantitative PCR (qPCR). DNA can be detected even after cell death, which makes RNA the better choice to study bacterial viability (Ndoye et al., 2011), except that RNA is extremely fragile and necessitates an additional step of retrotranscription to cDNA before further analysis. To avoid these problems, DNA can be used to study viability as long as its amplification in dead cells is blocked. Propidium monoazide (PMA) can now be used for this purpose (Nocker and Camper, 2006, 2009), but has never been used in cheese samples.

The aim of this study was to analyze probiotic viability during Cheddar cheese manufacture and ripening using a combination of qPCR and PMA-qPCR. Two strains of lactobacilli (*Lactobacillus helveticus* RO052 and *L. rhamnosus* RO011) and one strain of bifidobacteria (*B. animalis* subsp. *lactis* BB-12) were studied in single and mixed cultures. PMA-qPCR is optimized for cheese and its efficiency is compared with traditional culture media selective for each species.

## Materials and Methods

### Bacterial strains

*Lactobacillus helveticus* RO052 and *L. rhamnosus* RO011 were obtained in lyophilized or frozen form, respectively, from the Rosell-Lallemand Institute (Montreal, QC, Canada). Toxicity studies for these two strains have been completed and no toxicity was observed (Foster et al., 2011). *B. animalis* subsp. *lactis* BB-12 was obtained from Chr. Hansen (Hørsholm, Denmark) in frozen form. This culture, which has been used for over 25 years in fermented dairy products, has been classified as GRAS (Generally Recognized As Safe) by the FDA (Rulis, 2002) and approved by the Danish Medicines Agency as a Natural Health Product. The lactococci starter 970 (also in frozen form) was obtained from Fromagex (Rimouski, QC, Canada). All other bacterial species used for qPCR probe and primer specificity assessment were either obtained from the NCIMB collection (National Collection of Industrial, Marine and Food Bacteria, UK), the American Type Culture Collection (ATCC, Manassas, VA, USA), or were isolated from Canadian Cheddar cheese samples (Table 1).

**Table 1:** **Genomic DNA and 16S rDNA, *tuf* and *tal* sequences used for primer design and testing**.

Bacterial species and strain	Reference/source	16S rDNA gene accession number	*tuf* accession number	*tal* accession number
*Lactococcus lactis* subsp. *cremoris* SK11	Marakova et al. (2006)	4434136	4433356	4434063
*Lactococcus lactis* subsp. *lactis* IL1403	Bolotin et al. (2001)	1115962	1113987	1114937
*Bifidobacterium animalis* subsp. *lactis* BB-12	Chr. Hansen	GU116483	GQ302843	FJ357031
*Lactobacillus helveticus* RO052	Rosell-Lallemand Institute	DQ123580	DQ123584	NA**
*Lactobacillus rhamnosus* RO011	Rosell-Lallemand Institute	RO011_ r07923	RO011_02150	RO011_05652
*Lactobacillus casei* ATCC 334	Marakova et al. (2006)	4421666	4421117	4420558
*Lactobacillus parabuchneri*	Experimental Cheddar cheese	JQ247532*	NA**	NA**
*Lactobacillus brevis*	Experimental Cheddar cheese	JQ247531*	NA**	NA**
*Lactobacillus coryniformis*	Experimental Cheddar cheese	JQ247530*	NA**	NA**
*Lactobacillus curvatus*	Canadian commercial Cheddar cheese	JQ247525*	NA**	NA**
*Lactobacillus plantarum* ATCC 14917	American type culture collection	AF080101	NA**	NA**
*Lactobacillus zeae* ATCC 15820	American type culture collection	AB008213	NA**	NA**

*Submitted to GenBank after partial 16S rDNA gene sequencing to confirm identification.

**NA, no sequence available (DNA used to verify amplification efficiency).

### Cheesemaking procedures and chemical analysis

Experimental Cheddar cheese samples were manufactured following standard procedures (Kosikowski, 1977) in 10 L vats (INRA, Poligny, France). Briefly, whole milk was obtained from the Deschambault Animal Sciences Research Center and treated the same day by high temperature short time (HTST) pasteurization, followed by cooling at 32°C. Four repetitions of each type of cheese were randomly made for the first experiment, and three were made for the second experiment. The purpose of the first experiment was to optimize the quantification methods, and the cheese samples were manufactured with a 0.03% inoculation (based on microbiological counts) of the lactococci starter culture (starter 970 from Fromagex) and with a single culture of probiotic (RO052, RO011, or BB-12) inoculated after 5 min at an appropriate concentration to reach log 8 cfu/g of cheese before ripening (0.01% inoculation for RO052 and BB-12 and 0.0143% inoculation for RO011). The control cheese did not contain any probiotic adjunct (CTL). For the second experiment, inoculations were adjusted to higher levels to match traditional cheesemaking standards for lactococci starters (0.3% inoculation to reach log 9 cfu/g of cheese before ripening and Health Canada guidelines for probiotic consumption (0.1% inoculation for RO052 and BB-12 and 0.143% inoculation for RO011 to reach log 9 cfu/30 g portion of cheese before ripening). These cheese samples were manufactured with one to three probiotic strains [BB-12+RO052 (MC1), RO011+RO052 (MC2), BB-12+RO011+RO052 (MC3)], and included a control cheese without probiotics (MC0). A CaCl_2_ solution (0.02% v/v) was added, as was veal rennet (0.02% v/v; Fromagex), when the milk acidity had increased by 4.0°Dornic. When coagulation was achieved, the curd was cut and heated to 38°C, followed by whey draining and cheddaring. The resulting cheese was cut, salted (2.0% w/w) and pressed overnight to produce 1 kg blocks, which were then vacuum-packed in plastic bags and stored at 4°C. Microbiological and molecular analyses were conducted on samples taken after the initial probiotic inoculation, after cooking, after cheddaring, after salting, and after pressing. During ripening, new cheese samples were analyzed every month for a total of 4 (for samples MC0, MC1, MC2, and MC3) to 6 months (for samples CTL, BB-12, RO011, and RO052).

Total titratable acidity was measured at all cheesemaking stages using an automatic titrator (Sigma-Aldrich, Oakville, ON, Canada; data not shown) along with pH, and final pH of the cheese samples was also measured. All other chemical analyses were conducted on cheese samples after 7 days. Fat content was analyzed following the Mojonnier extraction method (Atherton and Newlander, 1977), and protein content, with the Dumas nitrogen combustion method (Wiles et al., 1998). Final humidity was calculated from dry matter determination, and salt content was calculated with Quantab strips (Quantab, Hach, Loveland, CO, USA). Sugar and organic acid concentrations were obtained through HPLC analysis (HPLC Waters 600, Waters Corp, Milford, MA, USA) using an ION-300 column (Transgenomic Inc., Omaha, NE, USA) with a mobile H_2_SO_4_ phase (0.02 N) and a 0.4 mL/min flow rate for chromatographic separation.

### Bacterial isolation from cheese samples

Ten grams of cheese were mixed with 90 mL of sterile 2% trisodium citrate (2% w/v) and homogenized with the Stomacher^®^ 400 Circulator (Seward, Worthing, West Sussex, UK) for 5 min at 230 rpm, two 1 mL samples of the resulting suspension were used, one for microbiological counts and the other for DNA extraction after PMA treatment.

### Bacterial enumeration using microbiological counts

One milliliter of homogenized cheese suspension was serially diluted in 9 mL of sterile peptone water (0.1%) acidified with cysteine (0.05%) and adjusted to a final pH of 6.8. For all three probiotic strains, the MRS culture medium (Fluka, Saint-Louis, MO, USA) was used in combination with a specific antibiotic for each strain. RO052 was grown on MRS agar containing 5 mg/L of ciprofloxacin (Fluka) and incubated in anaerobic jars for 72 h at 37°C. For RO011, 100 mg/L of vancomycin (EMD, USA) was used, and plates were incubated aerobically for 72 h at 43°C. Finally, 50 mg/L of mupirocin (Sigma, Saint-Louis, MO, USA) was used for BB-12 selection, and all plates were incubated at 37°C for 72 h in anaerobic jars. The M17 culture medium (Difco Laboratories, Detroit, MI, USA) was used for lactococci enumeration and incubated aerobically at 30°C for 48 h. Additionally, routine quality controls for total mesophilic and psychrotrophic bacteria (Plate Count Agar, EMD Millipore Corporation, Billerica, MA, USA), coliforms (Coliform plate 50, 3M, London, ON, Canada), and staphylococci (Baird Parker Agar, Oxoid, Hampshire, United Kingdom) were carried out on raw and pasteurized milk and on the fresh cheese samples (day one; data not shown).

### Propidium monoazide treatment

Bacterial suspensions were treated with PMA according to the procedure developed by Nocker and Camper (2006) with the following modifications made for cheese samples. One milliliter of the cheese suspension was centrifuged at 10,000×*g* for 20 min, and the fat layer was removed with sterile swabs before the cell pellet was suspended in 500 μL of a TE 2X solution (20 mM Tris HCl pH 8.0, 2 mM EDTA). The PMA powder (phenanthridium, 3-amino-8-azido-5-[3-(diethylmethylammonio) propyl]-6-phenyl dichloride, Biotium Inc., Hayward, CA, USA) was initially dissolved in 20% dimethyl sulfoxide (DMSO) to reach a final concentration of 20 mM and stored at −20°C. For each bacterial suspension, 1.25 μL of the PMA was added (final concentration of 50 μM), and the tubes were shaken in the dark for 5 min to allow for maximal PMA contact with DNA. Then, all tubes were placed in a PMA lamp apparatus (halogen 500 W, Ingenia Biosystems, Barcelona, Spain) for 15 min. The PMA-treated cell suspensions were then centrifuged at 10,000×*g* for 15 min to recover cell pellets for DNA extraction and stored at −20°C.

### DNA extraction

Total genomic DNA from cheese samples was obtained following the protocol from Licitra et al. (2007). Briefly, the DNeasy Blood and Tissue kit with the Gram positive bacteria DNA extraction protocol (Qiagen, Mississauga, ON, Canada) was used with the following modifications. Bacterial pellets were suspended in 180 μL (for pure cultures) or 400 μL (for cheese samples) of buffer for enzymatic lysis [20 mM Tris HCl pH 8.0, 2 mM EDTA, 1.2% Triton X-100, 20 mg/mL lysozyme (Sigma-Aldrich), 10 μL/ml of 5 U/mL mutanolysin (Sigma-Aldrich)] and incubated at 37°C for 1 h. Protein digestion was performed by adding 25 μL of proteinase K and 200 μL of AL buffer from the DNeasy Blood and Tissue kit (Qiagen) and incubating at 70°C for 30 min. The suspensions were then transferred to a 2 mL microtube containing 0.3 g of zirconium beads (1 mm diameter, Biospec Products, Bartlesville, OK, USA). Tubes were shaken twice for 90 s in a Mini-Beadbeater-16 (Biospec Products) and then centrifuged at 10,000×*g* for 10 min at room temperature. Finally, the nucleic acids were precipitated from the supernatant by adding 200 μL of ice-cold absolute ethanol. DNA purification was performed as specified in Qiagen’s instructions. Residual RNA was removed using DNAse free RNase (Roche, Laval, QC, Canada) according to the manufacturer’s instructions. DNA samples were stored at −20°C.

### Quantification of bacterial viability in PMA-treated samples

#### Primer and probe design for probiotic and lactococci detection

Primers for the 16S rRNA gene were designed for total lactococci, as well as for the elongation factor Tu gene (*tuf*) for *L. helveticus* and *L. rhamnosus* and for the transaldolase gene (*tal*) for *B. animalis* subsp. *lactis* (Table 2). For *L. rhamnosus*, a specific TaqMan^®^ probe was also designed for the *tuf* gene. Gene sequences for different *Bifidobacterium* sp., lactobacilli, and lactococci species were obtained from GenBank (Table 1) and aligned using Clustal W multiple sequence alignment software. Primers were manually designed using Primer Express software v. 2.0 (Applied Biosystems) using the sequence region with highest homology within the same species, but with high divergence between species. The PCR amplification was then simulated^1^, and specificity was verified *in silico* by BLASTN^2^. All primers were also tested for hairpins and primer dimers using the Oligo analyzer software 3.1 (Integrated DNA Technology).

**Table 2:** **qPCR primers, efficiency, and detection limits for all bacterial species**.

Bacterial species (selected gene)	Primers and probes*	Amplicon length (bp)	Concentration (μM/μL)	Slope	Intercept point	Efficiency (%)	*R*^2^	Range (copy number)	Detection limit (log cfu/g)
*Lactococcus* sp. (rDNA 16S)	F: 5′-GAGGCAGCAGTAGGGAATCTTC-3′ R: 5′-CTTGATGAGCTTTCCACTCTCA-3′	133	0.5 0.5	−3.4	44.9	99.4	0.99	10^4^–10^11^	5.0
*L. helveticus (tuf)*	F: 5′-TTACAAGGCGACAAGGAAGC-3′ R: 5′-CGACCTGAAGCAACAGTACC-3′	158	0.8 0.4	−3.3	41.3	101.9	0.99	10^3^–10^8^	5.0
*L. rhamnosus (tuf)*	F: 5′-CATGGCCCAATGCCACAA-3′ R: 5′-CAACGATGTATTCAACACCAACTT-3′ P:5′-CACGTGAACATATTCTGTTTGGCGCGT-3′	70	0.9 0.9 0.9	−3.3	45.5	99.9	0.99	10^3^–10^8^	5.9
*B. animalis* subsp. *lactis (tal)*	F: 5′-GCGCTGGGCTGCTCTGGAAGC-3′ R: 5′-TGGCGAGCTCATCGACATACT-3′	116	0.8 0.4	−3.3	46.3	99.6	0.99	10^4^–10^9^	5.0

*F, forward primer; R, reverse primer; P, probe.

#### Verification of primer specificity by quantitative PCR

The specificity of each primer pair to the targeted species was determined by qPCR against the genomic DNA of lactococci, lactobacilli, and bifidobacteria species, along with other LAB species commonly found in Cheddar cheese (Table 1). Each 20 μL PCR amplification contained 10 μL of Fast SYBR Green PCR Master Mix (or TaqMan 2X Master Mix, Applied Biosystems, for *L. rhamnosus*), 1 μL of DNA (50 ng/μL), and an optimized concentration of each primer (and probe for *L. rhamnosus*; Table 2). After SYBR Green amplification, a dissociation curve analysis was performed by increasing the temperature by 1°C every 20 s from 65 to 94°C to confirm the absence of non-specific amplification products.

#### Determination of PMA treatment efficiency

In order to confirm that PMA-qPCR only quantified viable cells, each bacterial species in defined dead to live cell ratio were quantified in pure culture and spiked into 1 g of sterilized cheese sample. Dead cells were obtained following the protocol by Taskin et al. (2011), either by heating cell suspensions to 100°C for 18 min in peptone water (for lactococci and RO011) or by adding isopropanol (for RO052 and BB-12) according to preliminary testing which indicated the best method and the adequate time period to kill the cells of each species without inducing DNA degradation. This experiment was carried out in duplicate. After PMA treatment, DNA was then extracted following the same protocol as the other cheese samples. qPCR amplifications were carried out in triplicate, and *C*_t_ results were then plotted against the corresponding microbiological count (cfu/g).

#### Detection limit and standard curves

To evaluate the detection limit of the PMA-qPCR protocol, serial dilutions (from 10^10^ to 10^4^ cfu/mL) of each bacterial species were spiked into 1 g of sterilized cheese sample and treated with PMA. DNA was then extracted following the same protocol as the experimental cheese samples. For the standard curves, the DNA from the highest bacterial concentration was serially diluted in nuclease-free water. qPCR amplifications were carried out in triplicate, and *C*_t_ results were then plotted against the corresponding microbiological count (cfu/g).

#### Quantitative PCR amplifications

Quantitative PCR assays were carried out in 96-well plates and performed in triplicate using an ABI PRISM 7500 Fast real-time PCR system with software version 2.0.1 (Applied Biosystems). Each 20 μL PCR amplification with SYBR Green primers contained 10 μL of Fast SYBR Green PCR Master Mix (Applied Biosystems), 1 μL of DNA, and an optimized concentration for each primer (Table 2). For RO011 quantification, 10 μL of the TaqMan 2X Master Mix (Applied Biosystems) were used, and 1 μL of the probe was added. A positive control of DNA (not treated with PMA) for each species and a no-template control (NTC) was included on each plate. Amplification consisted of a 20 s denaturation step at 95°C, followed by 40 two-step cycles of 3 s for 95°C and 30 s at 60°C. After amplification with the SYBR Green Master Mix, a dissociation curve analysis was performed. The cycle threshold of each sample was then compared to the corresponding PMA-treated DNA standard curve.

### Statistical analysis

Statistical analyses on viable counts and qPCR results were performed using the JMP9 Software (SAS Institute Inc). The Mixed procedure was conducted with a full factorial design (variance components covariance structure) with cheesemaking step, ripening time and single or mixed culture as fixed effects, and replicate cheese blocks as the random classification variable. When results were significantly different, a pairwise comparison of all means was done using the Tukey–Kramer HSD test.

## Results

### Optimization of PMA-qPCR for cheese

#### Cross amplification for primer and probe specificity

For all primer and probe sets designed to be specific for each targeted bacterial species (*Lactococcus* sp., *L. helveticus* RO052, *L. rhamnosus* RO011, and *B. animalis* subsp. *lactis* BB-12), *C*_t_ values for targeted DNA were between 17 and 21, and non-targeted DNA *C*_t_ values were always above 30 with no cross amplification (Table 3). Melting curves of amplicons only displayed one peak, also indicating specific amplification for each primer and probe set.

**Table 3:** **Specificity of primers and probe (*C*_t_ results) for gene quantification**.

Bacterial species and strain*	*Lactococcus* sp. (rDNA 16S)	*L. helveticus* RO052 *(tuf)*	*L. rhamnosus* RO011 *(tuf)*	*B. animalis* subsp. *lactis* BB-12 *(tal)*
*Lactococcus lactis* subsp. *cremoris* SK11	**17**	36	35	40
*Lactococcus lactis* subsp. *lactis* IL1403	**18**	36	36	40
*Bifidobacterium animalis* subsp. *lactis* BB-12	38	35	35	**18**
*Lactobacillus helveticus* RO052	38	**21**	31	33
*Lactobacillus rhamnosus* RO011	37	35	**17**	35
*Lactobacillus casei* ATCC 334	36	35	30	35
*Lactobacillus parabuchneri*	35	36	34	33
*Lactobacillus brevis*	36	36	35	35
*Lactobacillus coryniformis*	39	36	36	40
*Lactobacillus curvatus*	38	35	35	35
*Lactobacillus plantarum* ATCC 14917	36	34	36	34
*Lactobacillus zeae* ATCC 15820	37	34	33	33

*See Table 1 for GenBank accession numbers. Specific targeted DNA results are shown in bold letters*.

#### Quantitative PCR efficiency and detection limits

For qPCR, efficiencies for all primer and probe sets were between 99.4 and 101.9% with *R*^2^ values over 99% for all standard curves which covered at least six dilutions (Table 2). As for the detection limit in cheese samples (obtained by spiking), it was between 5 to 6 log cfu/g for all primer and probe sets, with the lowest value for *Lactococcus* sp., *B. animalis subsp. lactis*, and *L. helveticus* (5.0 log cfu/g) and the highest for *L. rhamnosus* (5.9 log cfu/g; Table 2).

#### Determination of propidium monoazide efficiency for viable cell quantification

In order to assess PMA efficiency in detecting only living cells and for blocking the quantification of dead cells, viable to dead cell ratios were obtained from pure cultures or spiked cheese samples and were submitted to qPCR with or without prior PMA treatment (Table 4). No significant difference was observed between results obtained from pure cultures or spiked cheese samples (*P* = 0.7062 for *Lactococcus* sp., *P* = 0.5352 for RO011, *P* = 0.3793 for RO052, and *P* = 0.1682 for BB-12), and quantification results decreased significantly with lower viable to killed cell ratios for PMA-treated samples (*P* = 0.0182 for *Lactococcus* sp., *P* = 0.0158 for RO011, *P* = 0.0149 for RO052, and *P* = 0.0091 for BB-12). For unblocked DNA samples (results obtained with no PMA treatment), quantification remained stable for all ratios with a slight but non-significant decrease for *Lactococcus* sp. and RO011 with lower viable to killed cell ratios.

**Table 4:** **Effect of propidium monoazide (PMA) on PCR quantification of defined ratios of viable to killed cells in pure culture or in Cheddar cheese samples**.

Bacterial species and strains	Viable to killed cell ratio (%)	Pure culture (log cfu/g)		Cheddar cheese samples (log cfu/g)	
		Without PMA	With PMA	Without PMA	With PMA
*Lactococcus* sp.	100	9.6 ± 1.7*	9.5 ± 0.9	9.7 ± 2.1	9.6 ± 1.7
	10	9.5 ± 1.8	8.8 ± 0.2	8.9 ± 0.2	8.7 ± 0.1
	1	9.2 ± 0.7	7.8 ± 0.1	9.0 ± 0.2	7.7 ± 0.1
	0.1	9.1 ± 0.6	6.9 ± 0.1	9.3 ± 0.1	6.6 ± 0.1

*L. helveticus* RO052	100	8.8 ± 0.1	8.9 ± 0.2	8.9 ± 0.1	8.7 ± 0.1
	50	8.9 ± 0.0	8.3 ± 0.1	9.0 ± 0.1	8.5 ± 0.1
	10	8.8 ± 0.2	7.5 ± 0.1	9.2 ± 0.6	7.5 ± 0.1
	5	8.7 ± 0.1	7.2 ± 0.1	9.0 ± 0.4	7.2 ± 0.1
	1	8.9 ± 0.0	7.0 ± 0.2	8.8 ± 0.1	6.8 ± 0.1

*L. rhamnosus* RO011	100	8.7 ± 0.7	8.9 ± 0.7	8.9 ± 1.9	8.8 ± 0.1
	50	8.6 ± 0.7	8.7 ± 2.1	8.8 ± 1.8	8.3 ± 0.3
	10	8.5 ± 0.6	8.3 ± 0.8	8.3 ± 0.1	8.0 ± 0.2
	5	8.5 ± 1.2	8.1 ± 0.4	8.4 ± 0.7	7.9 ± 0.3
	1	8.2 ± 0.3	7.5 ± 0.1	8.1 ± 0.2	7.7 ± 0.1

*B. animalis* subsp. *lactis* BB-12	100	9.5 ± 0.1	9.7 ± 0.1	9.4 ± 0.1	9.5 ± 0.1
	50	9.5 ± 0.1	9.2 ± 0.5	9.4 ± 0.1	9.1 ± 0.3
	10	9.8 ± 1.8	8.6 ± 0.2	9.4 ± 0.1	8.5 ± 0.1
	5	9.8 ± 0.9	8.4 ± 0.1	9.6 ± 0.8	8.0 ± 0.1
	1	9.7 ± 1.6	7.8 ± 0.2	9.6 ± 0.1	7.7 ± 0.2

*All results are expressed as mean values followed by their standard deviation (SD).

### Quantification of viability

Cultivable and viable cells were quantified with selective culture media and qPCR coupled with PMA (Tables 5 and 6). For *Lactococcus* sp. (Table 5), cultivable counts showed a one to two log increase until the cheddaring and salting stages, reaching log 9 cfu/g of cheese when probiotics were added in single culture, and log 10 cfu/g of cheese when they were added in mixed culture. Cultivable lactococcal counts then decreased up to six logs with ripening time (*P* < 0.0001) with significantly different results between cheese samples (*P* < 0.0001), the lowest viable counts being obtained in samples containing RO052 in single culture or combined with RO011 (MC2) or all three probiotic strains (MC3). PMA-qPCR results for all species produced significantly higher viable counts than cultivable counts obtained with culture media (*P* < 0.0001) as they reached log 12 cfu/g of cheese for lactococci during manufacture (Table 5). Viable counts did not decrease as much as cultivable counts with ripening time (*P* < 0.0001), as only a two to three log cfu/g of cheese decrease was observed, particularly when RO052 was added in single culture, but not when it was added in mixed culture.

**Table 5:** **Quantification (log cfu/g) *of Lactococcus* sp. in all cheese samples during cheesemaking and ripening using culture media and PMA-qPCR**.

Method	Cheese sample^†^	Cheesemaking steps					Ripening time (days)^††^					
		Inoculation	Cooking	Cheddaring	Salting	Pressing	30	60	90	120	150	180
Culture medium	CTL^1^	7.4 ± 0.1^c^*	8.6 ± 0.05^ab^	9.3 ± 0.1^a^	9.1 ± 0.1^a^	8.7 ± 0.2^a^	8.8 ± 0.1^ab^	8.5 ± 0.2^b^	7.6 ± 0.2^cd^	7.5 ± 0.1^cde^	7.1 ± 0.2^de^	6.8 ± 0.2^e^
	RO052^h^	7.5 ± 0.04^c^	8.6 ± 0.1^ab^	9.2 ± 0.03^a^	9.2 ± 0.2^a^	9.0 ± 0.1^a^	8.0 ± 0.2^ab^	7.4 ± 0.0^b^	5.6 ± 0.3^cd^	5.2 ± 0.3^cde^	5.4 ± 0.6^de^	4.8 ± 0.3^e^
	RO011^i^	7.4 ± 0.r	8.6 ± 0.1^ab^	9.2 ± 0.04^a^	9.2 ± 0.2^a^	9.1 ± 0.1^a^	8.8 ± 0.1^ab^	8.3 ± 0.2^b^	7.5 ± 0.1^cd^	7.1 ± 0.3^cde^	6.9 ± 0.2^de^	6.5 ± 0.2^e^
	BB-12^1^	7.4 ± 0.03^c^	8.6 ± 0.1^ab^	9.1 ± 0.1^a^	9.2 ± 0.2^a^	9.1 ± 0.1^a^	8.8 ± 0.02^ab^	8.4 ± 0.2^b^	8.1 ± 0.3^cd^	7.8 ± 0.2^cde^	7.4 ± 0.2^de^	7.5 ± 0.4^e^
	MC0^k^	9.0	9.9 ± 0.4^z^	10.4 ± 0.6^z^	10.1 ± 0.5^z^	10.1 ± 0.0.4^yz^	9.8 ± 0.3^wx^	9.6 ± 0.2^vw^	8.6 ± 0.1^uv^	8.6 ± 0.3^uv^	NA**	NA
	MC1^1^	8.7 ± 0.3^xy^	10.5 ± 0.1^z^	10.7 ± 0.2^z^	10.5 ± 0.02^z^	10.0 ± 0.1^yz^	6.9 ± 0.4^wx^	6.5 ± 0.4^vw^	5.5 ± 0.6^uv^	6.1 ± 0.3^uv^	NA	NA
	MC2^1^	9.0 ± 0.04^xy^	10.5	10.7 ± 0.2^z^	10.6 ± 0.1^z^	10.1 ± 0.3^yz^	7.0 ± 0.1^wx^	6.1 ± 0.2^vw^	5.5 ± 0.2^uv^	4.9 ± 0.3^uv^	NA	NA
	MC3^1^	9.0 ± 0.1^xy^	10.6	10.7 ± 0.2^z^	10.4 ± 0.1^z^	10.1 ± 0.2^yz^	7.1 ± 0.1^wx^	6.5 ± 0.3^vw^	5.4 ± 0.1^uv^	5.0 ± 0.2^uv^	NA	NA

PMA-qPCR	CTL^ij^	7.4 ± 0.3^a^	11.1 ± 1.1^efg^	10.9 ± 0.1^g^	10.6 ± 0.1^fg^	10.4 ± 0.1^efg^	10.3 ± 0.1^ef^	9.9 ± 0.1^de^	9.4 ± 0.2^cd^	9.1 ± 0.1^bc^	8.7 ± 0.2^ab^	8.6 ± 0.3^ab^
	RO052^h^	8.5 ± 0.6^a^	10.1 ± 0.1^efg^	10.8 ± 0.1^g^	10.6 ± 0.03^fg^	10.3 ± 0.1^efg^	10.0 ± 0.03^ef^	9.3 ± 0.1^de^	8.7 ± 0.3^cd^	8.2 ± 0.4^bc^	7.8 ± 0.6^ab^	7.8 ± 0.4^ab^
	RO011^hi^	7.7 ± 0.2^a^	10.0 ± 0.1^efg^	10.9 ± 0.1^g^	10.6 ± 0.1^fg^	10.3 ± 0.1^efg^	10.3 ± 0.1^ef^	9.8 ± 0.2^de^	9.1 ± 0.3^cd^	8.7 ± 0.3^bc^	8.4 ± 0.4^ab^	8.1 ± 0.4^ab^
	BB-12^j^	7.9 ± 0.2^a^	10.2 ± 0.03^efg^	10.9 ± 0.1^g^	10.7 ± 0.04^fg^	10.3 ± 0.1^efg^	10.3 ± 0.1^ef^	10.l ± 0.l^de^	9.6 ± 0.3^cd^	9.3 ± 0.2^bc^	9.1 ± 0.3^ab^	8.9 ± 0.4^ab^
	MCO^k^	9.1 ± 0.4^v^	11.6 ± 0.2^w^	12.2 ± 0.2^w^	11.9 ± 0.1^w^	11.5 ± 0.1^w^	11.3 ± 0.3^uv^	10.4 ± 0.2^uv^	9.4 ± 0.2^u^	9.4 ± 0.3^u^	NA	NA
	MCl^l^	8.8 ± 0.2^v^	11.8 ± 0.2^w^	12.2 ± 0.2^w^	11.7 ± 0.04^w^	11.2 ± 0.1^w^	10.0 ± 0.1^uv^	9.3 ± 0.3^uv^	9.0 ± 0.3^u^	9.0 ± 0.2^u^	NA	NA
	MC2^l^	9.5 ± 0.3^v^	11.6 ± 0.1^w^	12.0 ± 0.2^w^	11.8 ± 1.0^w^	11.7 ± 0.3^w^	9.4 ± 0.2^uv^	9.2 ± 0.1^uv^	8.6 ± 0.1	8.4 ± 0.1^u^	NA	NA
	MC3^l^	10.4 ± 0.6^v^	11.6 ± 0.2^w^	11.5 ± 0.6^w^	11.6 ± 0.2^w^	11.2 ± 0.1^w^	9.6 ± 0.1^uv^	9.1 ± 0.1^uv^	8.7 ± 0.1^u^	8.8 ± 0.3^u^	NA	NA

*^†^CTL, control for the single culture batch; BB-12, *B. animalis* subsp. *lactis* BB-12 in single culture; RO011, *L. rhamnosus* RO011 in single culture; RO052, *L. helveticus* RO052 in single culture; MCO, control cheese for the mixed culture batch; MCl, RO052+BB-12; MC2, RO052+RO011; MC3, RO052+RO011+BB-12*.

*^a–g^Results for single culture cheese samples not horizontally connected with the same letter indicate a significant difference between cheesemaking and ripening times (Tukey–Kramer HSD test; *P* < 0.05)*.

*^u–z^Results for mixed culture cheese samples not horizontally connected with the same letter indicate a significant difference between cheesemaking and ripening times (Tukey–Kramer HSD test; *P* < 0.05)*.

*^h–j^Results for single culture cheese samples not vertically connected with the same letter indicate a significant difference between samples (Tukey–Kramer HSD test; *P* < 0.05)*.

*^k, l^Results for mixed culture cheese samples not vertically connected with the same letter indicate a significant difference between cheese samples (Tukey–Kramer HSD test; *P* < 0.05)*.

*All results are means followed by their standard deviation (SD).

**NA, not analyzed for this point.

**Table 6:** **Quantification (log cfu/g) of each probiotic species in all cheese samples during cheesemaking and ripening using culture media and PMA-qPCR**.

Method	Quantified species	Cheese sample^†^	Cheesemaking steps					Ripening time (days)				
			Inoculation	Cooking	Cheddaring	Salting	Pressing	30	60	90	120	150	180
Culture Media	*L. rhamnosus*	RO0ll	6.5 ± 0.02^a^*	6.8 ± 0.2^ab^	7.0 ± 0.2^abc^	7.1 ± 0.2^abc^	7.8 ± 0.1^cd^	8.0 ± 0.1^d^	7.7 ± 0.2^cd^	7.5 ± 0.1^bcd^	7.5 ± 0.2^cd^	7.5 ± 0.1^bcd^	7.5 ± 0.1^bcd^	
		MC2^k^	8.2 ± 0.04^u^	9.1 ± 0.03^u^	9.3 ± 0.1^v^	9.1 ± 0.1^v^	9.5 ± 0.1^v^	9.3 ± 0.1^v^	9.4 ± 0.1^v^	9.3 ± 0.1^v^	9.2 ± 0.2^v^	NA**	NA
		MC3^k^	8.4 ± 0.1^u^	8.8 ± 0.4^u^	9.4 ± 0.02^v^	9.2 ± 0.1^v^	9.5 ± 0.2^v^	9.3 ± 0.2^v^	9.4 ± 0.1^v^	9.4 ± 0.2^v^	9.8 ± 0.3^v^	NA	NA
	*L. helveticus*	RO052	6.9 ± 0.3^ab^	7.1 ± 0.1^ab^	7.6 ± 0.03^b^	7.8 ± 0.l^b^	8.1 ± 0.l^b^	7.9 ± 0.2^b^	7.8 ± 0.3^b^	7.5 ± 0.0^b^	7.3 ± 0.l^b^	7.4 ± 0.2^b^	6.7 ± 0.2^ab^
		MCl^k^	8.5 ± 0.1^u^	9.2 ± 0.02^vwx^	9.5 ± 0.02^x^	9.4 ± 0.1^vwx^	9.6 ± 0.1^x^	9.5 ± 0.1^vwx^	9.4 ± 0.04^wx^	9.2 ± 0.2^vwx^	8.8 ± 0.2^uv^	NA	NA
		MC2^k^	8.5 ± 0.1^u^	9.3 ± 0.1^vwx^	9.5 ± 0.05^x^	9.4 ± 0.03^vwx^	9.6 ± 0.1^x^	9.4 ± 0.1^vwx^	9.7 ± 0.1^wx^	9.3 ± 0.1^vwx^	8.9 ± 0.2^uv^	NA	NA
		MC3^k^	8.7 ± 0.1^u^	9.3 ± 0.1^vwx^	9.6 ± 0.1^x^	9.3 ± 0.1^vwx^	9.9 ± 0.3^x^	9.5 ± 0.01^vwx^	9.4 ± 0.1^wx^	9.5 ± 0.2^vwx^	9.0 ± 0.1^uv^	NA	NA
	*B. animalis* subsp. *lactis*	BB-12	7.1 ± 0.1^a^	7.5 ± 0.l^b^	8.0 ± 0.1^c^	8.2 ± 0.03^c^	8.3 ± 0.1^c^	8.3 ± 0.03^c^	8.2 ± 0.1^c^	8.0 ± 0.l^bc^	8.0 ± 0.l^bc^	8.0 ± 0.1^c^	7.9 ± 0.l^bc^	
		MCl^k^	8.7 ± 0.02^u^	9.4 ± 0.02^vwx^	9.8 ± 0.1^wx^	10.1 ± 0.3^x^	9.7 ± 0.2^wx^	9.5 ± 0.2^vw^	9.5 ± 0.1^vw^	9.0 ± 0.3^uv^	8.9 ± 0.3^uv^	NA	NA
		MC3^k^	8.7 ± 0.03^u^	9.5 ± 0.1^vwx^	9.8 ± 0.1^wx^	10.0 ± 0.3^x^	9.8 ± 0.2^wx^	9.3 ± 0.1^vw^	9.4 ± 0.04^vw^	9.0 ± 0.1^uv^	8.9 ± 0.2^uv^	NA	NA

PMA-qPCR	*L. rhamnosus*	RO0ll	5.7 ± 0.2^a^	7.4 ± 0.02^bc^	7.9 ± 0.1^cd^	7.8 ± 0.1^cd^	8.1 ± 0.1^d^	7.1 ± 0.2^b^	7.4 ± 0.l^bc^	7.1 ± 0.2^b^	7.3 ± 0.l^bc^	7.2 ± 0.02^b^	7.4 ± 0.l^bc^	
		MC2^k^	6.4 ± 0.4^u^	8.1 ± 0.1^v^	8.2 ± 0.2^vw^	8.3 ± 0.2^vw^	8.6 ± 0.2^w^	8.6 ± 0.1^w^	8.5 ± 0.1^w^	8.5 ± 0.1^w^	8.6 ± 0.1^w^	NA	NA
		MC3^k^	6.7 ± 0.2^u^	7.8 ± 0.1^v^	8.5 ± 0.1^vw^	8.4 ± 0.2^vw^	8.6 ± 0.1^w^	8.7 ± 0.1^w^	8.5 ± 0.1^w^	8.5 ± 0.1^w^	8.5 ± 0.1^w^	NA	NA
	*L. helveticus*	RO052	5.2 ± 0.2^a^	7.1 ± 0.2^b^	7.8 ± 0.2^vw^	7.9 ± 0.1^c^	8.3 ± 0.1^cd^	8.8 ± 0.03^d^	8.7 ± 0.1^d^	8.5 ± 0.1^d^	8.7 ± 0.1^d^	8.6 ± 0.01^d^	8.5 ± 0.1^d^
		MC1^k^	6.9 ± 0.3^u^	9.3 ± 0.1^v^	9.5 ± 0.2^vw^	9.5 ± 0.2^vwx^	9.8 ± 0.1^wx^	9.8 ± 0.1^wx^	9.9 ± 0.1^wx^	9.5 ± 0.1^wx^	9.9 ± 0.1^x^	NA	NA
		MC2^k^	7.8 ± 0.3^u^	9.3 ± 0.1^v^	9.5 ± 0.2^vw^	9.6 ± 0.2^vwx^	10.0 ± 0.1^wx^	9.7 ± 0.03^wx^	9.9 ± 0.1^wx^	9.9 ± 0.1^wx^	10.0 ± 0.1^x^	NA	NA
		MC3^k^	7.7 ± 0.3^u^	9.1 ± 0.1^v^	9.6 ± 0.1^vw^	9.6 ± 0.1^c^	9.8 ± 0.04^wx^	9.7 ± 0.1^wx^	9.8 ± 0.1^wx^	9.9 ± 0.1^wx^	10.0 ± 0.1^x^	NA	NA
	*B. animalis* subsp. *lactis*	BB-12	6.0 ± 0.2^a^	8.2 ± 0.l^b^	8.9 ± 0.1^c^	9.0 ± 0.04^c^	9.1 ± 0.1^c^	9.1 ± 0.1^c^	9.2 ± 0.1^c^	8.8 ± 0.2^bc^	8.6 ± 0.l^bc^	8.8 ± 0.1^c^	8.7 ± 0.2^bc^	
		MC1^k^	7.5 ± 0.02^u^	10.3 ± 0.1^vwx^	10.6 ± 0.1^x^	10.5 ± 0.04^x^	10.5 ± 0.1^x^	10.5 ± 0.1^vwx^	10.3 ± 0.03^vwx^	9.9 ± 0.2^v^	10.0 ± 0.3^vw^	NA	NA
		MC3^k^	8.8 ± 0.2^u^	10.1 ± 0.1^vwx^	10.8 ± 0.1^x^	10.6 ± 0.05^x^	10.6 ± 0.1^x^	10.3 ± 0.04^vwx^	10.1 ± 0.2^vwx^	9.7 ± 0.1^v^	9.7 ± 0.2^vw^	NA	NA

*†CTL, control for the single culture batch; BB-12, *B. animalis* subsp. *lactis* BB-12 in single culture; RO011, *L. rhamnosus* RO011 in single culture; RO052, *L. helveticus* RO052 in single culture; MCO, control cheese for the mixed culture batch; MC1, RO052+BB-12. MC2, RO052+RO011; MC3, RO052+RO011+BB-12*.

*^a–g^Results for single culture cheese samples not horizontally connected with the same letter indicate a significant difference between cheesemaking and ripening times (Tukey–Kramer HSD test; *P* < 0.05)*.

*^u–x^Results for mixed culture cheese samples not horizontally connected with the same letter indicate a significant difference between cheesemaking and ripening times (Tukey–Kramer HSD test; *P* < 0.05)*.

*^h–j^Results for single culture cheese samples not vertically connected with the same letter indicate a significant difference between samples (Tukey-Kramer HSD test; *P* < 0.05)*.

*^k,l^Results for mixed culture cheese samples not vertically connected with the same letter indicate a significant difference between cheese samples (Tukey–Kramer HSD test; *P* < 0.05)*.

*All results are means followed by their standard deviation (SD).

**NA, not analyzed for this time point.

For probiotic strain viability (Table 6), no significant difference was found between mixed probiotic strain cheese samples. Using selective culture media, a one log increase in cfu/g of cheese was detected during cheesemaking, followed by a stabilization of cultivable counts during ripening for all species. Viable counts by PMA-qPCR were significantly lower than cultivable cell counts obtained with specific culture media at the inoculation point (*P* < 0.0001 for RO011, *P* = 0.0007 for RO052, and *P* < 0.0001 for BB-12), but reached higher levels afterward and were up to one log cfu/g cheese higher at the end of ripening (Table 6). All three probiotic strains appeared stable in all cheese samples during ripening.

### Chemical composition of the cheese samples

Final pH, moisture level, fat and protein content, as well as salt, glucose and lactic acid concentration were not significantly different between cheese samples of the same batch (made either with one probiotic strain or with a mixed probiotic culture; Table 7). However, salt-to-moisture ratios were significantly higher for cheese samples made with both lactobacilli strains (MC2; *P* = 0.0136), and acetic acid and galactose concentrations were significantly different between samples (*P* < 0.0001).

**Table 7:** **Chemical composition of each cheese sample after 14 days of ripening at 4°C**.

Component	Cheese sample^†^							
	CTL	BB-12	RO011	RO052	MCO	MCI	MC2	MC3
Final pH	5.2 ± 0.1^a^*	5.1 ± 0.13^a^	5.2 ± 0.2^a^	5.1 ± 0.2^a^	5.3 ± 0.1^x^	5.2 ± 0.2^x^	5.2 ± 0.2^x^	5.3 ± 0.1^x^
Fat (%)	29.2 ± 4.6^a^	26.6 ± 3.6^a^	29.0 ± 4.9^a^	30.8 ± 3.2^a^	33.7 ± 3.9^x^	33.5 ± 4.6^x^	33.3 ± 4.2^x^	33.0 ± 4.2^x^
Moisture (%)	40.1 ± 0.6^a^	39.8 ± 1.5^a^	39.5 ± 1.8^a^	39.6 ± 0.7^a^	41.2 ± 0.7^x^	43.7 ± 7.9^x^	38.8 ± 1.7^x^	44.5 ± 9.6^x^
Salt (%)	1.3 ± 0.1^a^	1.3 ± 0.08^a^	1.3 ± 0.1^a^	1.2 ± 0.2^a^	1.4 ± 0.07^x^	1.5 ± 0.3^x^	1.9 ± 0.5^x^	1.8 ± 0.1^x^
Salt-to-moisture (%)	3.2 ± 0.3^a^	3.2 ± 0.2^a^	3.3 ± 0.3^a^	3.2 ± 0.6^a^	3.3 ± 0.8^x^	3.4 ± 0.5^x^	4.9 ± 0.4^x^	3.9 ± 0.7^x^
Protein (%)	22.6 ± 0.3^a^	22.3 ± 0.4^a^	22.5 ± 0.3^a^	22.7 ± 0.2^a^	22.4 ± 1.2^x^	23.4 ± 1.3^x^	23.0 ± 1.0^x^	23.6 ± 0.1^x^
Lactose (%)	0.4 ± 0.04^a^	0.4 ± 0.05^a^	0.4 ± 0.02^a^	0.2 ± 0.02^b^	0.4 ± 0.00^x^	0.3 ± 0.00^x^	0.2 ± 0.00^x^	0.3 ± 0.00^x^
Galactose (%)	0.03 ± 0.01^a^	0.05 ± 0.03^ab^	0.01 ± 0.00^a^	0.1 ± 0.01^b^	0.03 ± 0.00^xy^	0.06 ± 0.00^z^	0.03 ± 0.00^x^	0.04 ± 0.00^y^
Glucose (%)	0.0 ± 0.0^a^	0.0 ± 0.0^a^	0.0 ± 0.0^a^	0.0 ± 0.0^a^	0.0 ± 0.1^x^	0.0 ± 0.0^x^	0.0 ± 0.0^x^	0.0 ± 0.0^x^
Lactic acid (%)	1.0 ± 0.01^a^	1.0 ± 0.02^a^	0.9 ± 0.03^a^	1.0 ± 0.06^a^	0.5 ± 0.00^x^	0.6 ± 0.00^x^	0.7 ± 0.00^x^	0.5 ± 0.00^x^
Acetic acid (%)	0.04 ± 0.02^a^	0.05 ± 0.02^a^	0.01 ± 0.00^a^	0.04 ± 0.03^a^	0.0 ± 0.0^x^	0.01 ± 0.00^y^	0.01 ± 0.00^y^	0.01 ± 0.00^y^

*†CTL, control for the single culture batch; BB-12, *B. animalis* subsp. *lactis BB-12* in single culture; RO0ll, *L. rhamnosus RO*011 in single culture; RO052, *L. helveticus* RO052 in single culture; MCO, control cheese for the mixed culture batch; MCI, RO052+BB-12. MC2, RO052+RO011; MC3, RO052+RO011+BB-12*.

*^a, b^Results for single culture cheese samples not horizontally connected with the same letter indicate a significant difference (Tukey–Kramer HSD test; *P* < 0.05)*.

*^x, y^Results for mixed culture cheese samples not horizontally connected with the same letter indicate a significant difference (Tukey–Kramer HSD test; *P* < 0.05)*.

*All results are means followed by their standard deviation (SD).

## Discussion

In fermented food products such as cheese, synergy between all bacterial species present must be maintained without any negative effect of probiotic addition on product quality, especially when more than one probiotic strain is used. The impact of the bacterial ecosystem and the cheese environmental conditions (pH, *A*_w_, redox potential, and nutrient availability) on probiotic strains must also be verified (Champagne et al., 2005; Saarela et al., 2006). The aim of this study was to assess the viability of three probiotic strains added in single or mixed culture in Cheddar cheese samples ripened for up to 6 months by comparing the efficiency of microbiological and molecular approaches.

The selective culture media used for this study were specific to each species, but their analysis was very time consuming and do not correctly estimate viable cell counts during cheese ripening as the antibiotics created restrictive culture conditions (Rawsthorne and Phister, 2009). Also, some cells may be in an uncultivable state while still contributing enzymatic activities (Lahtinen et al., 2006; Coutard et al., 2007). The use of PMA-qPCR is better suited to quantify total cellular viability (Kramer et al., 2009; Nocker and Camper, 2009) and was optimized to work in Cheddar cheese samples, as DNA from dead cells was efficiently blocked by the PMA molecules. PMA-qPCR has already been used to quantify probiotics in yogurt (García-Cayuela et al., 2009), but has not been used in cheese to date.

In this study, lactococci from the starter culture decreased faster during ripening when probiotic strains were present, especially when *L. helveticus* RO052 was added. The presence of a combination of strains did not cause a greater reduction in viable lactococci counts than single strains. This could be explained by the lower pH values and concomitant non-dissociated organic acids in cheese samples containing probiotics, although no significant pH difference was found. Cultivable counts using culture media greatly underestimated total lactococci viability when compared to PMA-qPCR results, indicating the presence of many VBNC cells in ripening Cheddar cheese which can only be detected by using molecular methods. Dolci et al. (2010) also detected dominant lactococci in cheese samples using DNA analysis, although they were not detected in such numbers with microbiological methods. Rantsiou et al. (2008) and Flórez et al. (2006) proposed that the presence of active lactococci throughout cheese ripening was higher than previously believed. These bacteria can still maintain their metabolic activity even though they cannot be cultivated and can therefore contribute to the development of Cheddar cheese texture and flavor during ripening (Ndoye et al., 2011).

The probiotic strains were chosen for their commercial use in fermented food products such as cheese and yogurt, *L. helveticus* RO052 and *L. rhamnosus* RO011 originating from dairy products (Hagen et al., 2010; Foster et al., 2011). Both strains are resistant to acidic pH levels (Dimitrov et al., 2005; Succi et al., 2005) which are known to negatively affect probiotic viability, especially during dairy product storage (Champagne et al., 2005). They also have a higher tolerance to bile salts (Kheadr, 2006) and have been found in the intestine after ingestion (Verdu et al., 2008), supporting the requisite attributes for a probiotic strain. *B. animalis* subsp. *lactis* BB-12 has also been widely studied and used in various probiotic food products because of its resistance to higher acidity levels and its greater oxygen tolerance (Lee and O’Sullivan, 2010).

In this study, all probiotic strains in mixed culture cheese samples remained at a 10^9^ cfu/g level throughout ripening according to viable counts obtained by selective culture media, a level which is thought to be sufficient to offer a health benefit (Karimi et al., 2011). Cheese is already known as an efficient food carrier for probiotics because of its high fat content and its high buffering capacity which enhance bacterial survival through the intestinal track (Dinakar and Mistry, 1994). Some studies report sufficient survival of probiotic strains in Cheddar cheese (Phillips et al., 2006; Ong et al., 2007), but others were not as successful (Lynch et al., 1996; Gardiner et al., 1998) depending on the choice of strains. Their interaction with the starter, which could have a negative impact by its metabolic activity (increase in acidity levels, competition for nutrients, production of antagonistic components) could also impact probiotic strains negatively, but starter cultures can also degrade milk compounds into new nutrients and lower the oxygen concentration in the environment to the benefit of probiotics (Heller, 2001; Champagne et al., 2005). Adding the probiotic strains directly to milk, and not during cheesemaking, is also known to increase their viability (Fortin et al., 2011). Furthermore, as higher salt concentration is known to be detrimental to probiotic viability (Fortin et al., 2011), the lower salt content in our samples, which was often below the targeted 1.5%, could have contributed to probiotic survival in our study. The ripening temperature used (4°C) could also have contributed to the stability of viable counts (Sanders and Huis in’t Veld, 1999; Heller, 2001; Champagne et al., 2008).

Results obtained by PMA-qPCR were lower than those obtained by traditional microbiological methods for the first data point, indicating that culture media may lack specificity and detect non-targeted probiotic strains or non-starter lactic acid bacteria naturally found in Cheddar cheese, especially with lower numbers of viable probiotic cells at the beginning of cheesemaking. However, the viable counts obtained by PMA-qPCR reached higher levels of log 10 cfu/g during cheesemaking. As results obtained by molecular methods are more accurate than those obtained by traditional microbiological methods, acceptance of PMA-qPCR for viable bacteria quantification could be helpful for cheese manufacturers in adjusting inoculation level and reducing production cost, as less viable cells would actually be required during the cheesemaking process in order to achieve the legislated level of log 9 cfu/30 g portion during storage. PMA-qPCR quantification for viability assessment showed greater accuracy for probiotic quantification in yogurt as well (García-Cayuela et al., 2009). However, although all primers were designed to be specific to the chosen lactobacilli and bifidobacterium species, the presence of non-probiotic strains of the same species in cheese samples (especially *L. rhamnosus* and *L. helveticus*) might augment the quantification of strains with health benefits.

For all probiotic strains used in our study, there was no significant impact of strain interaction on their viable counts with either quantification method, indicating no ill-effect on viability when the strains were used together in the Cheddar cheese matrix. These strains could therefore be combined in a probiotic cheese, which should widen the health benefits of such a product (Matto et al., 2006; Oksaharju et al., 2011). A combination of *L. rhamnosus* RO011 and *L. helveticus* RO052 has already been tested in capsule form, demonstrating their basic synergy (Foster et al., 2011), but their health benefits in interaction with *B. animalis* subsp. *lactis* BB-12 remain to be studied. The use of multiple probiotic strains as adjunct could also contribute to accelerated cheese ripening and enhance flavor through their effect on lactococci as well as through their own metabolic activity (Ong et al., 2007).

To our knowledge, this study was the first to optimize PMA-qPCR to monitor probiotic viability in Cheddar cheese. The results demonstrate that PMA-qPCR is a specific and powerful approach compared with traditional quantification methods for the three probiotic species as well as for lactococci in cheese. The technique can be used with mixtures of species and provides an accurate estimation of viability in cheese. This approach could now be used to further assess bacterial survival in other types of cheese and to determine the impact of probiotic strain interactions on such food matrices.

## Conflict of Interest Statement

The authors declare that the research was conducted in the absence of any commercial or financial relationships that could be construed as a potential conflict of interest.

## Footnotes

^1^http://insilico.ehu.es/PCR/

^2^http://blast.ncbi.nlm.nih.gov/
